# Role of the ERK pathway in psychostimulant-induced locomotor sensitization

**DOI:** 10.1186/1471-2202-7-20

**Published:** 2006-03-02

**Authors:** Emmanuel Valjent, Jean-Christophe Corvol, James M Trzaskos, Jean-Antoine Girault, Denis Hervé

**Affiliations:** 1 INSERM, U536, F-75005, Paris; Université Pierre et Marie Curie (UPMC-Paris 6), F-75005, Paris; Institut du Fer a Moulin, F-75005, Paris; 2 Bristol-Myers Squibb Co., Pharmaceutical Research Institute, Princeton, NJ 08540, USA

## Abstract

**Background:**

Repeated exposure to psychostimulants results in a progressive and long-lasting facilitation of the locomotor response that is thought to have implications for addiction. Psychostimulants and other drugs of abuse activate in specific brain areas extracellular signal-regulated kinase (ERK), an essential component of a signaling pathway involved in synaptic plasticity and long-term effects of drugs of abuse. Here we have investigated the role of ERK activation in the behavioral sensitization induced by repeated administration of psychostimulants in mice, using SL327, a brain-penetrating selective inhibitor of MAP-kinase/ERK kinase (MEK), the enzyme that selectively activates ERK.

**Results:**

A dose of SL327 (30 mg/kg) that reduced the number of activated ERK-positive neurons by 62 to 89% in various brain areas, had virtually no effect on the spontaneous locomotor activity or the acute hyperlocomotion induced by cocaine or D-amphetamine. Pre-treatment with SL327 (30 mg/kg) prior to each drug administration prevented the locomotor sensitization induced by repeated injections of D-amphetamine or cocaine. The SL327 pre-treatment abolished also conditioned locomotor response of mice placed in the context previously paired with cocaine or D-amphetamine. In contrast, SL327 did not alter the expression of sensitized response to D-amphetamine or cocaine.

**Conclusion:**

Altogether these results show that ERK has a minor contribution to the acute locomotor effects of psychostimulants or to the expression of sensitized responses, whereas it is crucial for the acquisition of locomotor sensitization and psychostimulant-conditioned locomotor response. This study supports the important role of the ERK pathway in long-lasting behavioral alterations induced by drugs of abuse.

## Background

Behavioral sensitization corresponds to a progressive enhancement of locomotor responses following repeated exposure to cocaine or D-amphetamine (D-amph) [1]. When established, sensitization is long-lasting since it is observed after re-exposure to the drug several weeks or even one year later [2]. Sensitization is thought to underlie important aspects of vulnerability to drug addiction and relapse [2,3]. In rodents sensitization was shown to enhance predisposition to psychostimulant self-administration [4] and to facilitate the reinstatement by drugs of extinguished self-administration [5,6]. Behavioral sensitization is strengthened by association of psychostimulant injections with contextual cues and context-dependent sensitization involves different behavioral and neurobiological mechanisms from context-independent sensitization [7,8].

Processes underlying induction and expression of behavioral sensitization involve a complex interplay between various neurotransmitters and neuromodulators including dopamine, glutamate (see [9,10]), neuropeptides and trophic factors [11-14]. It can be hypothesized that these converging extracellular signals give rise to a limited number of specific molecular and cellular events that mediate behavioral sensitization to psychostimulants. Several lines of evidence indicate the involvement of the ERK pathway in the integration of extracellular signals and in the long-term effects of drugs of abuse [15,16]. ERK is activated in reward-associated brain areas (including nucleus accumbens (NAcc), dorsal striatum, amygdala and prefrontal cortex, ventral tegmental area (VTA) through combined stimulation of dopamine and glutamate receptors after acute or repeated treatment with psychostimulant drugs [15-21]. In the NAcc, activated ERK controls the state of phosphorylation of transcription factors including Elk1 and cAMP response element binding protein (CREB) and, thereby, initiates a gene transcription program that is supposed to lead to the long-term effects of repeated exposure to psychostimulants [22]. However, although the role of the ERK pathway in the rewarding properties of various drugs is well established [15,23-25], its role in locomotor sensitization induced by repeated drugs administration is not characterized.

In the present study we analyzed the involvement of the ERK pathway in the locomotor responses induced by acute and also repeated administration of psychostimulants. Our results show that blockade of the ERK pathway by the MEK inhibitor SL327 has limited effects on the acute locomotor responses to cocaine or D-amph, but prevents the induction of sensitization induced by repeated administration of these drugs, as well as the conditioned locomotor responses in the environment previously paired with drug injection.

## Results

### Inhibition of ERK phosphorylation in the brain by systemic injection of SL327

To evaluate the role of the ERK pathway in the behavioral responses to psychostimulants, we used systemic administration of the MEK inhibitor SL327 that crosses the blood-brain barrier [26]. We first evaluated the efficacy of SL327 to inhibit MEK in the brain by counting the number of neurons immunopositive for diphospho-ERK (P-ERK) in several brain areas involved in the addictive effects of psychostimulants [18] (Table 1). SL327 (15, 30 and 50 mg/kg, i.p.) dose-dependently reduced the number of P-ERK-positive neurons with a similar potency in all the regions. A significant inhibition of ERK activation was observed in all the analyzed regions of mice pre-treated with 30 mg/kg of SL327. The inhibition was complete with 50 mg/kg, as indicated by the absence of P-ERK labeling.

### Role of the ERK pathway in spontaneous and acute D-amph- and cocaine-induced hyperlocomotion

**Figure 1:**
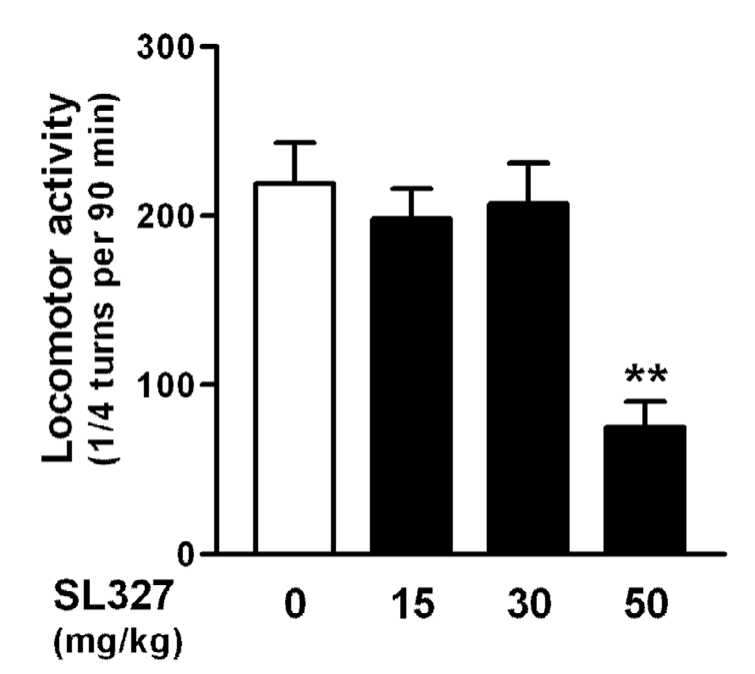
**Effects of SL327 on spontaneous locomotor activity**. Locomotor activity was measured during 90 min after acute administration of vehicle or the MEK inhibitor SL327 (15, 30 and 50 mg/kg). Data were analyzed with one-way ANOVA (F_(3,92) _= 10.57, P < 0.001) followed by Bonferroni test (** P < 0.01, SL327 vs vehicle). 24 mice per group.

The effects of SL327 on spontaneous locomotor activity of mice were measured during 90 min. As illustrated in Figure 1, acute injection of SL327 at doses that significantly inhibited ERK activation (15, 30 mg/kg, Table 1) had no effect on the basal locomotor activity. However, at a higher dose (50 mg/kg), SL327 reduced by about 60% the spontaneous locomotor activity (Fig. 1).

**Figure 2:**
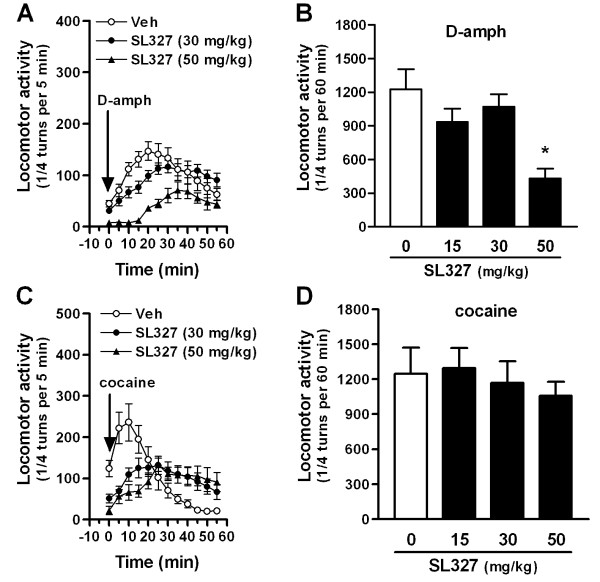
**Effects of SL327 on the locomotor activity induced by injection of D-amph or cocaine**. Mice pre-treated with vehicle or SL327 (15, 30 and 50 mg/kg) were injected 30 min later with D-amph (2 mg/kg i.p., **A**, **B**) or cocaine (20 mg/kg i.p., **C**, **D**). **A**) Time course of D-amph-induced locomotor activity measured every 5 min is illustrated for mice pre-treated by Vehicle (Veh) or the highest doses of SL327 (30 mg/kg or 50 mg/kg) (10 mice per group). Time course obtained with 15 mg/kg SL327 that was similar to that obtained with 30 mg/kg SL327 is not shown for improving legibility of the figure. Data (30 and 50 mg/kg SL327 and vehicle) were analyzed with a mixed factor ANOVA (repeated measure over time) (effect of SL327 F_(2,216) _= 43.55, P < 0.01). **B**) Total locomotor activity during the first hour after D-amph administration was analyzed with one-way ANOVA (F_(3,36) _= 5.277, P < 0.01). * P < 0.01 Vehicle (Veh) vs SL327 (50 mg/kg). **C**) Time course of cocaine-induced locomotor activity measured every 5 min for mice pre-treated by Vehicle (Veh) or SL327 (30 mg/kg or 50 mg/kg) (10 mice per group). Time course obtained with 15 mg/kg SL327 was similar to that obtained with 30 mg/kg SL327 (not shown in the figure). Data (30 and 50 mg/kg SL327 and vehicle) were analyzed with a mixed factor ANOVA (repeated measure over time) (effect of SL327 F_(2,216) _= 12.745, P < 0.05). **D**) Total locomotor activity during the first hour after cocaine administration was analyzed with one-way ANOVA (F_(3,36) _= 1.058, NS).

We investigated the consequences of pre-treatment with various doses of SL327 on the locomotor effects of D-amph (2 mg/kg, i.p.). SL327 at dose of 15 and 30 mg/kg slightly delayed the locomotor hyperactivity induced by D-amph and diminished the amplitude of the peak response (Fig. 2A, and data not shown). However, the duration of locomotor hyperactivity was longer than in vehicle-treated mice, and the total counts of locomotion during a 60 min period (area under the curve after drug injection) were not altered at the doses of 15 and 30 mg/kg (Fig. 2B). In contrast, pre-treatment with 50 mg/kg SL327, markedly inhibited D-amph-induced hyperlocomotion during the first 30 min after drug administration (Fig. 2A) leading to a significant decrease in the total counts of D-amph-induced locomotion for 60 min (Fig. 2B).

Similar experiments were carried out using cocaine (Fig. 2C, D). The time course of cocaine locomotor responses measured every 5 min was modified by SL327 pre-treatment at doses of 15, 30 and 50 mg/kg: it was delayed and prolonged in the presence of SL327 and the peak response was diminished (Fig. 2C, D). However, the hyperlocomotion had a longer duration and, therefore, the total locomotor activity induced by cocaine over a 1-hour period was not altered by SL327 pre-treatment (Fig. 2D).

### Role of the ERK pathway in the development of locomotor sensitization induced by D-amph

**Figure 3:**
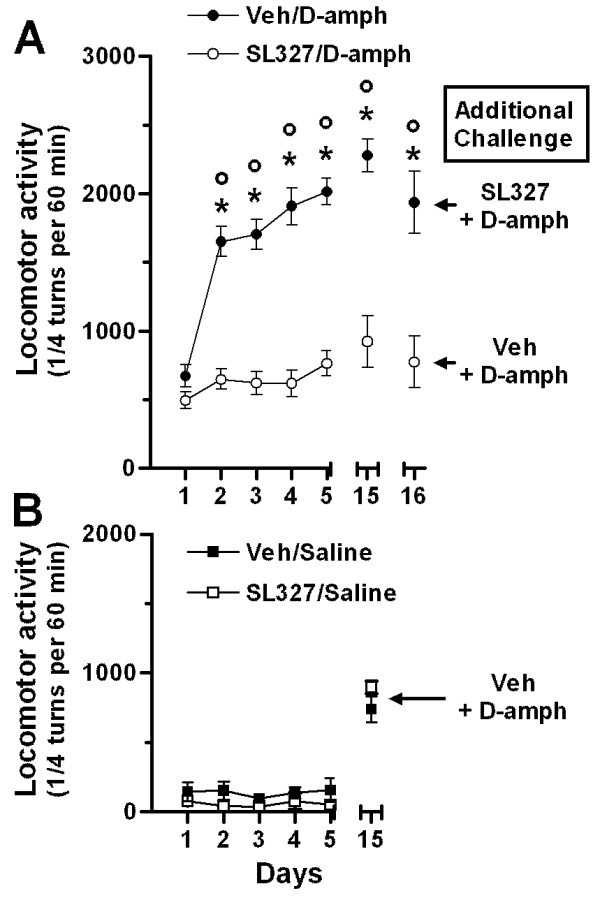
**Effects of SL327 on the induction and expression of locomotor sensitization by repeated administration of D-amph**. A) Locomotor responses were measured during 5 consecutive days after administration of D-amph (2 mg/kg, i.p.) 30 min after pre-treatment with vehicle (Veh) or SL327 (30 mg/kg), as indicated (10 mice per group). After 10 days of withdrawal (day 15), the Veh/D-amph and SL327/D-amph groups received an additional challenge with Veh/D-amph or SL327/D-amph, respectively. The following day (day 16, Additional Challenge), mice treated previously with Veh/D-amph were challenged with D-amph in the presence of SL327 whereas those treated previously with SL327/D-amph received an injection of D-amph alone. B) Locomotor responses measured during 5 consecutive days after administration of saline 30 min after pre-treatment with vehicle (Veh) or SL327 (30 mg/kg), as indicated (10 mice per group). After 10 days of withdrawal (day 15), the Veh/saline and SL327/saline groups received an additional treatment with Veh/D-amph. Data were analyzed with a mixed factor ANOVA (repeated measure over time): for saline group (Days 1–5: effect of time: F_(4,90) _= 2.15, NS; effect of SL327: F_(1,90) _= 1.07, NS); for D-amph group (Days 1–15: effect of time: F_(6,126) _= 13.48, P < 0.01; effect of SL327: F_(1,126) _= 26.21, P < 0.01). Bonferroni test: * P < 0.01 compared to day 1, ° P < 0.01 Veh/D-amph vs SL327/D-amph.

Since 30 mg/kg SL327 blocked efficiently ERK activation and induced only a limited alteration of the acute locomotor response to D-amph we used this dose for examining the effects of ERK blockade on the development of behavioral sensitization to psychostimulants. Four groups of mice were injected daily for 5 consecutive days in the actimeter. Mice received D-amph or saline 30 min after pre-treatment with SL327 or vehicle (see Materials and Methods). The locomotor response of mice treated with D-amph without SL327 pre-treatment enhanced progressively, providing evidence of the expected behavioral sensitization (Fig. 3A). By contrast, when SL327 (30 mg/kg) was injected 30 min before each D-amph administration, the locomotor response remained similar during the five days (Fig. 3A). Thus, the two groups of D-amph-treated mice, with and without SL327 pre-treatment, had a similar locomotor response the first day, but displayed significant differences the following days (Fig. 3A). In saline-injected mice, the locomotor activity was similar after SL327 or vehicle pre-treatment and remained stable during the five days (Fig. 3B).

After a 10-day period of withdrawal, the maintenance of behavioral sensitization was evaluated (day 15). As expected, in the group of mice pre-exposed to D-amph without SL327 the challenge with D-amph produced a locomotor response similar to that on day 5 indicating the maintenance of sensitization (Fig. 3A). In the group of mice pre-exposed to D-amph in the presence of SL327, a challenge with D-amph in the presence of SL327 did not reveal any enhancement of locomotor response on day 15, suggesting the complete absence of locomotor sensitization by SL327/D-amph treatment. However, there was a possibility that SL327/D-amph-treated mice developed a sensitization to D-amph but did not express it because the D-amph challenge was also preceded by the SL327 treatment. If it were the case, blockade of the ERK pathway by SL327 would not impair the induction of locomotor sensitization but only its expression. To test this possibility, an additional challenge with D-amph without SL327 pretreatment was performed on day 16 in the group of SL327/D-amph-treated mice. This challenge produced a low locomotor response similar to that on day 1 showing clearly the absence of induction of sensitization to D-amph in this group of mice. Thus, the blockade of the ERK pathway by SL327 altered the induction of sensitization. We examined whether this blockade affected also its expression. To test this possibility, the mice which had received chronic injections (5 days) of D-amph alone and which were sensitized, as shown by their increased response to D-amphetamine on day 15 (Fig. 3A), were challenged with D-amph in presence of SL327 on day 16. The injection of D-amph in the presence of SL327 on day 16 produced a locomotor response similar to that induced by D-amph alone on day 15, revealing that blockade of the ERK pathway had no effect on the expression of behavioral sensitization.

As a control, we tested whether the repeated administration of SL327 could by itself alter the response to D-amph. A D-amph challenge was performed after the 10-day withdrawal period (day 15) in the mice chronically treated with vehicle/saline or SL327/saline. The locomotor response to D-amph in these groups was similar to that observed in mice receiving the first injection of D-amph (Fig. 3A, B) showing that chronic treatment by SL327 did not alter locomotor response to D-amph after 10-day withdrawal.

These results clearly showed that the D-amph-induced behavioral sensitization did not develop if the ERK pathway was blocked before each D-amph treatment. In contrast, when the animals were already sensitized by D-amph, ERK blockade did not affect the expression of sensitized response.

### Role of the ERK pathway in the development of locomotor sensitization induced by cocaine

**Figure 4:**
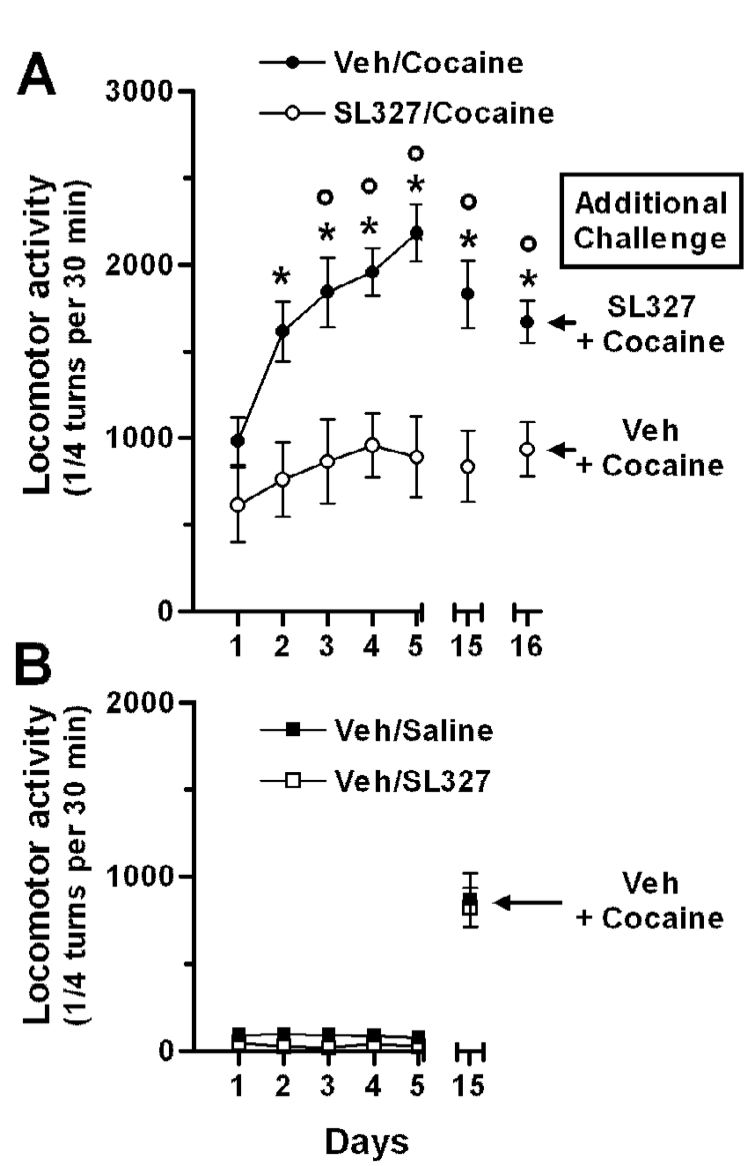
**Effects of SL327 on the induction and expression of locomotor sensitization by repeated administration of cocaine**. **A**) Locomotor responses measured during 5 consecutive days after administration of cocaine (20 mg/kg, i.p.) 30 min after pre-treatment with vehicle (Veh) or SL327 (30 mg/kg), as indicated (10 mice per group). After 10 days of withdrawal (day 15), the Veh/cocaine and SL327/cocaine groups received an additional treatment with Veh/cocaine and SL327/cocaine, respectively. **B**) Locomotor responses measured during 5 consecutive days after administration of saline 30 min after pre-treatment with vehicle (Veh) or SL327 (30 mg/kg), as indicated (10 mice per group). After 10 days of withdrawal (day 15), the Veh/saline and SL327/saline groups received an additional treatment with Veh/cocaine. Data were analyzed with a mixed factor ANOVA (repeated measure over time): for saline group (effect of time: F_(4,90) _= 1.66, NS; effect of SL327: F_(1,90) _= 1.29, NS); for cocaine group (effect of time: F_(6,126) _= 11.27, P < 0.01; effect of SL327: F_(1,126) _= 18.27, P < 0.01). Bonferroni test: * P < 0.01 compared to day 1, ° P < 0.01 Veh/D-amph vs SL327/D-amph.

Since repeated cocaine exposure also induces a strong locomotor sensitization, the protocol described above for D-amph was used to evaluate whether the blockade of the ERK pathway could affect cocaine-induced behavioral sensitization. As expected, the locomotor response of mice treated with cocaine without SL327 pre-treatment progressively enhanced, showing a clear behavioral sensitization (Fig. 4A). In contrast, when SL327 (30 mg/kg) was injected 30 min before each cocaine administration, the locomotor response remained similar during the five days (Fig. 4A).

After a 10-day period of withdrawal (day 15), we verified by a challenge with vehicle/cocaine that the sensitization was maintained in the group treated by repeated vehicle/cocaine. In contrast a challenge by SL327/cocaine did not reveal any sensitization in the group treated by repeated SL327/cocaine. On day 16, additional challenges were performed in these two groups of mice for investigating the respective effects of blockade of ERK pathway on the induction and expression of locomotor sensitization to cocaine. Our results were essentially similar to those obtained with D-amph. SL327 had no effect on the expression of sensitization, i.e. it did not decrease the sensitized locomotor response when it was administered just before the challenge injection (group vehicle/cocaine, challenge SL327/cocaine, day 16). In contrast, in mice that had received SL327 prior to each cocaine injection (group SL327/cocaine), the responsiveness to a challenge injection of cocaine alone (challenge vehicle/cocaine, day 16) was identical to that observed following the first injection, supporting the role of ERK in the induction of cocaine sensitization.

In the control groups treated with repeated vehicle/saline or SL327/saline, the injection of cocaine ten days after the end of chronic treatments (on day 15) produced locomotor responses similar to those observed in animals receiving the first injection of cocaine (Fig. 4A, B). This showed that chronic treatment by SL327 did not alter locomotor response to cocaine. Altogether, these results demonstrate that ERK is critical for the development, but not the expression, of the cocaine-induced behavioral sensitization.

### Role of the ERK pathway in the D-amph- or cocaine-conditioned locomotor response

**Figure 5:**
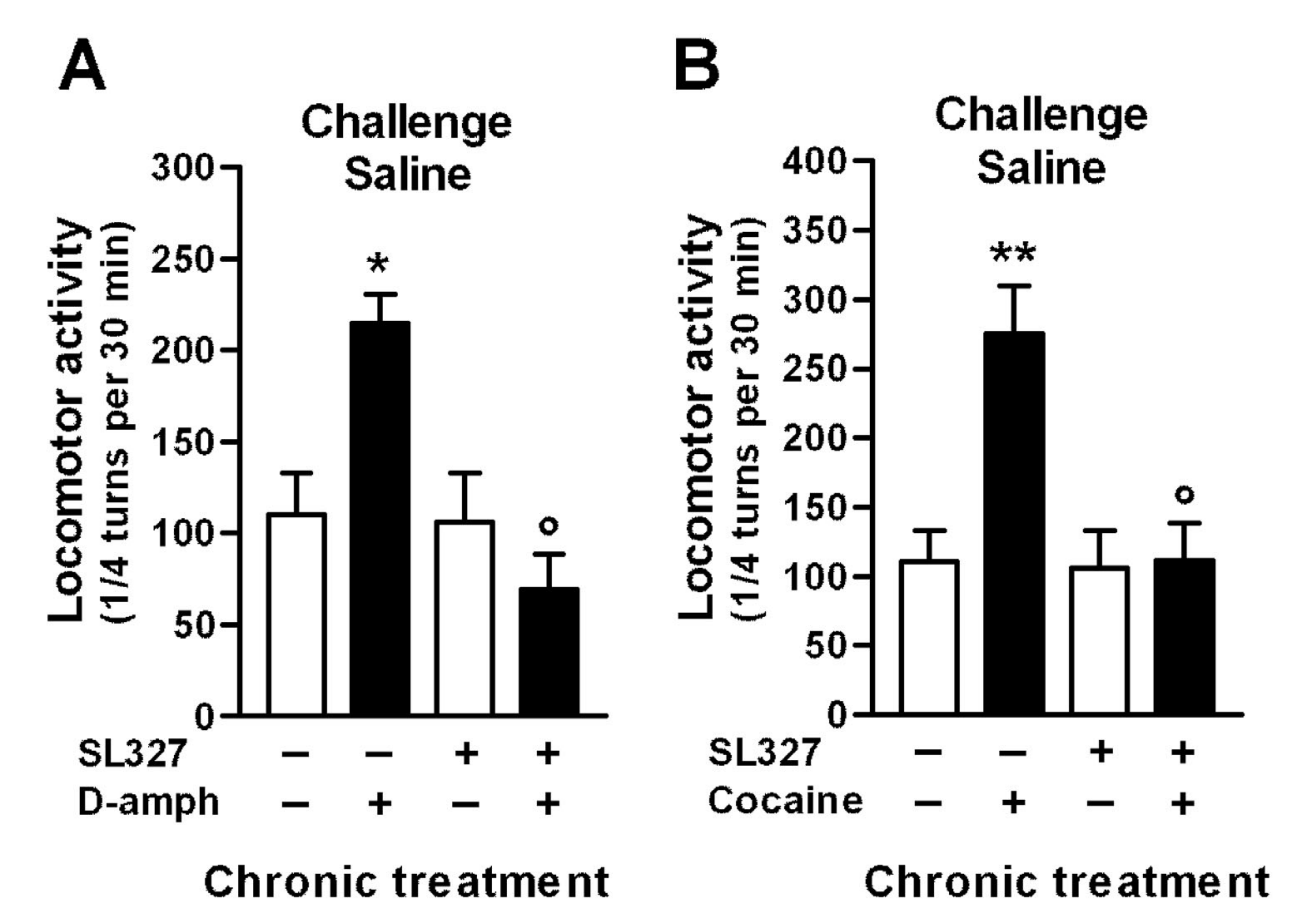
**Effects of SL327 on the induction of a conditioned locomotor response to saline administration**. **A**) Locomotor responses measured during the first 30 min after injection of saline in mice which have received repeated injections of saline or D-amph, preceded by pre-treatment by vehicle (Veh) or SL327 (30 mg/kg) 30 min before (see Fig 3). Saline injection was done in the same actimeter in which the mice had received the repeated injections. **B**). Analysis of conditioned response in mice chronically treated with cocaine (same protocol as in A except that mice had received repeated injections of cocaine, 20 mg/kg, instead of D-amph). Data were analyzed with one-way ANOVA (A): F_(3,36) _= 8.235, P < 0.001; (B) F_(3,36) _= 8.585, P < 0.001, followed by Bonferroni test: * P < 0.05, ** P < 0.01 saline vs D-amph or cocaine; ° P < 0.01 vehicle vs SL327. 10 mice per group.

During the protocol used for sensitizing mice to D-amph or cocaine, the mice developed association between context and drug effect. This was indicated by the conditioned locomotor response seen following saline injection in the environment associated with drug injections as compared to an environment associated with saline injection (Fig. 5). We examined whether SL327 pre-treatment before each drug injection during the conditioning phase could influence the drug-conditioned locomotor response. The conditioned response was completely abolished in mice pre-treated with SL327 before each injection of D-amph (Fig. 5A) or cocaine (Fig. 5B), demonstrating the critical role of ERK in this association.

## Discussion

### Role of ERK activation in behavioral sensitization to psychostimulants

This study provides evidence of the importance of ERK pathway in an important and characteristic long-term behavioral changes induced by drugs of abuse, locomotor sensitization. Drugs of abuse induce a rapid and strong activation of ERK in a precise and limited set of brain areas including NAcc, bed nucleus of stria terminalis, central amygdala, prefrontal cortex [15,18]. Two ERK isoforms, ERK1 and ERK2, are expressed in virtually every cell types. However, ERK2 appears more specifically implicated than ERK1 in the long-term effects of drugs since it is much more phosphorylated than ERK1 in the striatum following psychostimulant administration [16]. In addition, ERK1-deficient mice that displayed a stimulus-dependent increase in ERK2 signaling exhibited a potentiation of the rewarding properties of morphine in the conditioned place preference test (CPP) [27].

The strong activation of ERK induced by drugs of abuse raises the question of its functional role. The acute locomotor responses were only moderately affected by the pharmacological blockade of ERK pathway by systemic administration of the selective MEK inhibitor, SL327. The main effect of this inhibitor was a dose-dependent slowing of the effects of psychostimulants. By contrast, MEK blockade prevented the development, but not the expression, of the behavioral sensitization induced by repeated exposure to psychostimulants. These results strongly support the involvement of the ERK pathway in the delayed consequences of repeated psychostimulants administration, confirming and extending our previous finding that ERK activation was necessary for the induction of behavioral sensitization induced by a single injection of cocaine or D-amph [16].

### Role of different brain regions in ERK-dependent behavioral sensitization

Locomotor sensitization appears to involve several brain regions, most of which are the site of ERK activation by psychostimulants [9]. It has been previously reported that acute administration of psychostimulant (cocaine and D-amph) was able to produce an intense activation of ERK in neurons of the NAcc, lateral bed nucleus of the stria terminalis, central amygdale, deep layers of prefrontal cortex and VTA [18-21]. It is likely that the activation of ERK in these brain areas, or a subset of them, is critical for the induction of locomotor sensitization.

For both cocaine and D-amph, the induction of behavioral sensitization is thought to depend primarily on molecular events that occur in the VTA and promote a long-lasting increase in function of the meso-accumbens dopamine pathway [9]. The role of ERK in the VTA is supported by the report that local injection of a selective MEK inhibitor, PD98059, before each cocaine administration blocked the initiation of locomotor sensitization without altering the acute locomotor responses [12]. However, regulation of the ERK phosphorylation in response to psychostimulants is not as robust in the VTA as in the NAcc. Indeed, it has been reported that cocaine activated ERK in the VTA following repeated but not single injections [17,18] whereas D-amph activated ERK in the VTA following single but not repeated injections [21]. In addition, ERK activation by D-amph appears to occur only in non-dopaminergic neurons of the VTA [21]. Thus there is a possibility that the ERK activation in other areas could also contribute to the induction of behavioral sensitization.

We and others have found that psychostimulant administration produces intense ERK activation in a subset of medium size spiny neurons in the NAcc as well as in the dorsal striatum [15,16,19]. This regulation required combined action of dopamine D1 receptors and glutamate receptors [15,16,18-20] as well as the PKA-dependent phosphorylation of DARPP-32, an important regulatory protein of D1 receptor signaling cascade in the striatum, and the inhibition of protein phosphatase-1 [16]. In the NAcc, activation of the ERK pathway is the main factor responsible for the induction of Fos family genes (including c-fos, fosB and fra2), Zif268 and Egr3 in response to cocaine, D-amph and 3,4-methylenedioxymethamphetamine (MDMA) [15,19,20,24]. Since these genes encode transcription factors, ERK, by activating them, initiates a complex program of gene regulation that participates in the development of long-lasting effects of drugs of abuse [20,28,29]. However, the evidence supporting the implication of NAcc in the primary events leading to the induction of sensitization is relatively weak and limited to cocaine [30,31]. On the other hand, a number of neuroadaptations with possible consequences for behavioral responses were reported to occur in the NAcc following repeated exposure to psychostimulants. These neuroadaptations include functional supersensitivity of dopamine and AMPA receptors, changes in dopamine and glutamate release, decreased strength of cortico-striatal glutamate synapses, alteration in dendritic morphology (for review see [32]). Interestingly, some of these changes, such as the functional supersensitivity of NAcc dopamine D1 receptor, can be produced by repeated local infusion of D-amph into the VTA and appear secondary to an initial action of psychostimulants in the VTA [33]. ERK activation in the NAcc could contribute to the development of these secondary neuroplasticity events and thereby to alterations of NAcc neurons innervated by the VTA, which are necessary for the expression of sensitized response.

Finally, ERK activation in the prefrontal cortex may also be involved in locomotor sensitization. ERK is activated in the prefrontal cortex after cocaine or D-amph administration [18,34] and this could participate in the molecular adaptations leading to sensitization to cocaine or D-amph, since prefrontal cortex was shown to be essential for this effect [35-37]. Altogether, these observations suggest that the ERK pathway may play a role in the development of cocaine- and D-amph-induced behavioral sensitization at the level of VTA and possibly of other brain areas.

### Role of ERK activation in long term behavioral effects of drugs of abuse

Cocaine shares with other drugs commonly abused by humans, including morphine, nicotine and Δ9-tetrahydrocannabinol (THC), the property to activate ERK in the NAcc, lateral bed nucleus of the stria terminalis, central amygdala and deep layers of prefrontal cortex [18-21]. Other psychoactive compounds with less or no addictive potency (caffeine, scopolamine, antidepressants and antipsychotics) moderately activate ERK in some of these areas, but they do not induce this combined pattern of strong activation [18,38]. Thus, such a pattern of strong ERK activation appears to be the hallmark of drugs of abuse and suggests that ERK is involved in at least some of the common long term behavioral effects of these drugs. In support of this hypothesis, blockade of the ERK pathway, by systemic administration of SL327 or local inhibition in the NAcc, reduced the rewarding properties of cocaine, THC, amphetamine and MDMA evaluated by CPP [15,23-25,39]. Here we report that ERK blockade impairs the development of D-amph- or cocaine-induced conditioned locomotion. This locomotor response elicited by repeatedly pairing environmental context or stimuli with psychostimulant injections displays many similarities with classical pavlovian conditioning, environmental cues being the conditioned stimuli and drugs the unconditional stimulus [40,41]. This effect appears to result from a dopamine overflow in the NAcc following presentation of drug-associated stimuli [42]. Our data strongly suggest that ERK activity is required for the formation of conditioned associations between environmental stimuli and drug. Recently it has been reported that the activation of the ERK pathway in the central amygdala was critical for cue-controlled cocaine seeking in rats [43]. Altogether these results indicate that ERK activity is important for drug-induced pavlovian conditioning to environmental stimuli and for the acquisition by these stimuli of motivating properties. These behavioral processes are thought to be crucial for human addiction since exposure to environmental stimuli previously associated with drug-taking is a potent factor triggering drug craving and relapse in humans [44].

## Conclusion

The present study provides strong evidence that ERK activation is required for the development of behavioral sensitization induced by repeated administration of psychostimulants, as well as for drug-conditioned response. Altogether these results suggest that the ability to activate ERK in a specific set of brain regions is an important mechanism for the long lasting behavioral alterations induced by drugs of abuse that contribute to the development of addiction.

## Materials and methods

### Animals

Eight-week old C57BL/6J mice were from Charles River Laboratories, L'Arbresle, France. Animals were housed under constant temperature (22°C) and humidity (60%) with a constant cycle of 12 h light and 12 h dark and had free access to food and water. All experiments were performed in accordance with the guidelines of the French Agriculture and Forestry Ministry for handling animals (decree 87849, license 01499).

### Drugs

D-amphetamine sulfate (D-amph, 2 mg/kg) and cocaine hydrochloride (20 mg/kg) (Sigma-Aldrich, St Quentin Fallavier, France) were dissolved in saline solution (9 g/l NaCl in water). Mice received an intraperitoneal injection (20 ml/kg i.p.) of D-amph, cocaine or saline solution. SL327 (Bristol-Myers Squibb, Princeton, New Jersey, USA) was dissolved in 25% DMSO (vol/vol), diluted twice in water (final concentration of DMSO, 12.5%) and injected i.p. (10 ml/kg). Control mice received the same injection of diluted DMSO without SL327 (vehicle).

### Immunofluorescence

Thirty minutes after treatment with SL327 or vehicle, mice were rapidly anaesthetized with pentobarbital (30 mg/kg, i.p., Sanofi, France) and transcardially perfused with 4% (weight/vol.) paraformaldehyde in 0.1 M Na_2_HPO_4_/NaH_2_PO_4 _buffer (pH 7.5). Brains were post-fixed overnight in the same solution as used for perfusion and stored at 4°C. Sections (30 μm-thick) were cut with a vibratome (Leica, France) and kept at -20°C in a solution containing 30% (vol/vol) ethylene glycol, 30% glycerol (vol/vol) and 0.1 M phosphate buffer. Active ERK was immunolabeled with rabbit polyclonal antibodies against diphospho-ERK1/2 (1:400, Cell Signaling Technology) and Cy3-coupled secondary antibodies (1:400, Molecular Probes) as previously described [15]. Sections were mounted in Vectashield with DAPI counterstain (Vector laboratories). Images were obtained using sequential laser scanning confocal microscopy (SP2, Leica). Quantification was performed by counting the number of cells with a nuclear immunofluorescence above background using a computerized image analyzer, in 2–4 brain sections per animal, bilaterally for each region of interest.

### Behavioral analysis

#### Measurement of locomotor activity

Locomotor responses were evaluated using a circular corridor with four infrared beams placed at every 90° (Imetronic, Pessac, France) in a low luminosity environment, avoiding stress. Locomotor activity was counted when animals interrupted two adjacent beams and, thus, had traveled 1/4 of the circular corridor. Locomotor activity was measured by 5 min intervals and cumulative counts were taken for data analysis. Mice were habituated to the test apparatus (actimeter), handling, and procedure for three consecutive days before the actual experiments. In these 45-min sessions mice received saline injection 15 min after being placed in the apparatus.

#### Acute locomotor response

The acute locomotor effects of SL327 alone, or D-amph (2 mg/kg) or cocaine (20 mg/kg), with SL327 or vehicle pre-treatment were evaluated on day 4 (i.e. after the 3 habituation sessions) as followed: spontaneous activity during 15 min, locomotor activity after administration of vehicle or SL327 during 30 min and locomotor activity after injection of saline, D-amph or cocaine during 60 min.

#### Locomotor sensitization

##### Development of sensitization

For developing locomotor sensitization, mice were treated daily with saline, D-amph (2 mg/kg) or cocaine (20 mg/kg) for 5 consecutive days in the actimeter. Locomotor activity was measured as follows: spontaneous activity during 15 min, locomotor activity after administration of vehicle or SL327 (30 mg/kg) during 30 min and locomotor activity after injection of saline, D-amph or cocaine during 60 min.

##### Maintenance of locomotor sensitization and effect of SL327 on sensitization expression

The maintenance of locomotor sensitization was evaluated after 10 days of withdrawal (day 15) in the groups of mice repeatedly exposed to D-amph or cocaine. Those repeatedly treated in the absence of SL327 received a vehicle/D-amph (2 mg/kg) or vehicle/cocaine (20 mg/kg) treatment whereas those repeatedly treated in the presence of SL327 received a SL327/D-amph or SL327/cocaine treatment. In addition, control groups of mice that had repeatedly received saline injection in the presence or absence of SL327, were administered with a vehicle/D-amph or vehicle/cocaine treatment. Locomotor activity was measured using the same procedure as described above.

In order to test whether the blockade of the ERK pathway altered the expression of locomotor sensitization, the following day (day 16), the groups of mice previously exposed to psychostimulant in the absence of SL327 received a SL327 pre-treatment prior to a challenge injection of the corresponding psychostimulant. As a control, the groups of mice previously exposed to psychostimulant in the presence of SL327 received a vehicle pre-treatment prior to injection of the corresponding psychostimulant.

### Conditioned locomotor response

Groups of mice were treated daily with saline, D-amph (2 mg/kg) or cocaine (20 mg/kg) for 5 consecutive days in the actimeter as described above. The saline or psychostimulant injections were preceded 30 min before by a vehicle or SL327 (30 mg/kg) treatment. One day after the end of these treatments, all the mice were injected with saline in the drug-associated environment (i.e. the circular actimeter). Saline injections were done after a 30-min habituation period and the locomotor activity was recorded during 30 additional min after injection.

### Statistical analysis

Groups of 4 animals and at least 10 animals were used for immunofluorescence and behavior studies, respectively. Behavioral results Data were analyzed with one-way ANOVA, two-way ANOVA or mixed factor ANOVA (repeated measure over time), as required by the experimental design. Post-hoc analysis was done with Bonferroni test.

## Authors' contributions

EV carried out the behavioral and immunofluorescence studies, participated to the design of the studies and the statistical analysis of data, and drafted the manuscript. JCC participated to the behavioral experiments. JMT provided material necessary for experiments. JAG and DH conceived the study, participated in its design and coordination, and in revising the manuscript. All authors read and approved the final manuscript.

## Figures and Tables

**Table 1:** Effects of SL327 on ERK phosphorylation in various brain areas (% of vehicle-treated controls)

**Brain areas**		Vehicle	SL327 (15 mg/kg)	SL327 (30 mg/kg)	SL327 (50 mg/kg)	F_(3,12)_
Dorsal Striatum		100 ± 18	46 ± 10**	11 ± 6***	0 ± 0***	17.61
N. Accumbens	Core	100 ± 16	62 ± 15	13 ± 8**	0 ± 0***	15.55
	Shell	100 ± 13	65 ± 13	23 ± 12**	0 ± 0***	16.37
BNST	Lateral	100 ± 11	52 ± 5**	18 ± 7**	0 ± 0***	39.68
Amygdala	Central	100 ± 15	62 ± 11	15 ± 13**	0 ± 0***	16.15
Prefrontal Cortex		100 ± 10	63 ± 27	38 ± 17**	0 ± 0***	6.34

The number of positive nuclei was counted as described in Materials and Methods, and expressed as % of the average of controls (data are means ± SEM, 4 mice per group). BNST: bed nucleus of the stria terminalis. ANOVA F values are indicated in the right column (for all values P < 0.01). Post-hoc comparison of Vehicle vs SL327 was done with Bonferroni test. ** P < 0.01, *** P < 0.001.
